# New Technology for the Production of Transparent Glass Coatings from Multi-Alloy Targets with Antibacterial Activity

**DOI:** 10.3390/ma19010175

**Published:** 2026-01-03

**Authors:** Przemysław Ząbek, Jolanta Jaroszuk-Ściseł, Artur Nowak, Małgorzata Majewska, Anna Słomka, Agnieszka Hanaka, Ewa Ozimek, Radosław Swadźba, Maciej Liśkiewicz, Krzysztof Radwański

**Affiliations:** 1 D.A.Glass Sp. z o.o., Innowacyjna 15, 36-060 Głogów Małopolski, Poland; p.zabek@fibrain.pl; 2 Department of Industrial and Environmental Microbiology, Faculty of Biology and Biotechnology, Maria Curie-Skłodowska University, Akademicka 19, 20-031 Lublin, Poland; jolanta.jaroszuk-scisel@mail.umcs.pl (J.J.-Ś.); malgorzata.majewska@mail.umcs.pl (M.M.); asanna727@gmail.com (A.S.); ewa.ozimek@mail.umcs.pl (E.O.); 3 Department of Plant Physiology and Biophysics, Faculty of Biology and Biotechnology, Maria Curie-Skłodowska University, Akademicka 19, 20-031 Lublin, Poland; agnieszka.hanaka@mail.umcs.pl; 4 Materials Research Centre, Łukasiewicz Upper Silesian Institute of Technology, K. Miarki 12-14, 44-100 Gliwice, Poland; radoslaw.swadzba@git.lukasiewicz.gov.pl (R.S.); maciej.liskiewicz@polsl.pl (M.L.); krzysztof.radwanski@git.lukasiewicz.gov.pl (K.R.)

**Keywords:** magnetron-sputtering industrial line, target, multi-alloy, photosynthetically active radiation, transparent coatings, antibacterial, greenhouse temperature

## Abstract

**Highlights:**

**What are the main findings?**

- Cu-based sputtered films show strong antibacterial activity.
- Cu90Sn10, Cu90Zn10, and Cu80Ti20 targets give coatings with the best performance.
- Films exhibit high durability and corrosion resistance.
- Large-area multicomponent nanolayers were successfully produced.

**What are the implications of the main findings?**

- Cu-alloy targets are promising for greenhouse glass surfaces.
- Industrial PVD enables uniform large-area antimicrobial films.
- Findings support future antibacterial greenhouse applications.
- Scalable nanolayer design enables reproducible industrial coating.

**Abstract:**

Antibacterial thin-film coatings are of increasing interest for enhancing hygiene in controlled environments such as commercial greenhouses. Phytopathogens including *Pseudomonas syringae*, and human pathogens such as *Escherichia coli*, *Micrococcus luteus*, and *Staphylococcus aureus,* frequently contaminate greenhouse environments. The present study aimed to develop and evaluate multifunctional magnetron-sputtered glass coatings with strong antimicrobial performance, deposited by physical vapor deposition to achieve precise control of film composition and uniform coverage of large substrates (≥0.25 m^2^), ensuring industrial-scale applicability. Thin films were fabricated by magnetron sputtering using multi-alloy targets composed of Cu, Sn, Zn, Al, Ni, Fe, Ti, Mn, Nb, or Co. Fourteen distinct coating compositions were characterized using high-resolution transmission electron microscopy, scanning transmission electron microscopy, and energy-dispersive X-ray spectroscopy. Antibacterial performance was evaluated against the following strains: *E. coli* (PCM 2560), *M. luteus* (PCM 525), *S. aureus* (PCM 2602), and *P. syringae* pv. tomato (IOR2146). Coatings prepared from 90%Cu-10%Sn, 90%Cu-10%Zn, and 80%Cu-20%Ti targets exhibited one of the highest antibacterial efficiencies. These coatings also showed strong mechanical durability and corrosion resistance. Our results indicate that coatings obtained from Cu-based multi-alloy targets by magnetron sputtering are promising candidates for use as durable, antimicrobial inner glass surfaces in future greenhouse applications.

## 1. Introduction

The global commercial greenhouse market is expected to reach USD 72.51 billion by 2030, at a compound annual growth rate (CAGR) of 9.7% from 2022 to 2030 [1,2]. The demand for commercial greenhouses has grown rapidly in recent years, and it is expected to accelerate throughout the forecast period. Due to the population increase as well as climate changes, which impact crop output, the global commercial greenhouse market is being driven by increased food sales. In addition, an increasing awareness of commercial techniques to maximize yield will present a variety of market development prospects in the coming years [2].

Commercial greenhouses provide a healthy and highly regulated habitat. These conditions are very good for cultivating a variety of plants, including flowers, vegetables, and berries, as well as transplants. Regardless of the local climate, soil, or topography, greenhouses enable reliable plant growth. Thanks to their higher yields compared with traditional cultivation methods, greenhouses attract growing attention. Other factors, such as rapid urbanization, climate change, the expansion of sustainable agricultural systems, and finally, the rising demand for floriculture and ornamental horticulture, will likely accelerate the growth of the glass greenhouse market within the projected timeframe of 2021–2028. On the other hand, an increasing amount of research and development in this area, a rising demand for fruit and vegetables in growing economies, along with growing environmental concerns worldwide, will further contribute to generating immense opportunities and lead to the growth of the glass greenhouse market [3,4,5]. A very important factor influencing a high crop yield is a pathogen-free environment. Nowadays, this is achieved by using certain fungicides or pesticides, which unfortunately might affect human health [6]. This is why any solutions that reduce or eliminate the use of fungicides should be of the highest interest to every farmer [7].

Aware of this fact, D.A.Glass Sp. z o.o. has recently developed an antimicrobial glass with diffuse properties by means of a special coating (patent pending under number P.446040), which eliminates microorganisms existing on plants and surfaces in the greenhouse. This antimicrobial glass was obtained using the magnetron sputtering method, which is one of the physical vapor deposition (PVD) techniques that makes it possible to cover glass with a very thin, homogeneous layer of nanometer-range thickness (here: nanolayer). This method might be employed to apply thin films to various substrates, such as glass, silicon, metals, and polymers, for use in electronics, optics, and protective coatings [8,9,10,11]. The process relies on bombarding the surface of a material (the target) with ions of a gas (usually argon and/or oxygen) to eject atoms or molecules, which then are deposited on the substrate to form a transparent coating. A magnetic field is generated near the target, causing electrons to spiral, which increases the number of collisions between electrons and gas atoms. This leads to more efficient ionization of the gas and higher plasma density, consequently increasing the sputtering rate. This improves the efficiency of the process and enables the deposition of high-quality, uniform coatings [12,13]. It should be emphasized that, until now, the vast majority of studies have focused solely on laboratory-scale antibacterial and/or antifungal coatings produced using the magnetron sputtering method or its modifications, such as high-power pulsed magnetron sputtering (HPPMS)/direct current (DC) [14,15] and radiofrequency (RF) [16] magnetron sputtering.

Recently, antimicrobial coatings have gained significant attention in biomedical and healthcare environments due to their combined role as protective coatings and as functional layers that suppress microbial colonization and biofilm formation [16,17]. Most strains of the *Pseudomonas syringe* species are phytopathogens that infect a wide range of crop plants through natural orifices or wounds, with successful colonization and disease development facilitated, among other things, by surface adhesion [18,19,20]. *Escherichia coli*, *Micrococcus luteus,* and *Staphylococcus aureus* are common bacteria found in various environments (soil, water, or air), but they are not identified as phytopathogens [21]. However, in the human body, these microorganisms can cause dangerous diseases and damage tissues [22]. Such types of microorganisms settle on various structural elements of the greenhouse, as well as on the plants cultivated there, and are therefore perceived as bacteriological pollutants, which cause several foodborne diseases. For this reason, these species are considered in the antibacterial testing of coatings of materials used in food production, including in greenhouses [23].

One of the key mechanisms contributing to the antibacterial performance of engineered coatings is their surface hydrophobicity. This property plays a crucial role in the development of materials exhibiting antibacterial and anti-soiling properties. Hydrophobic coatings effectively repel water, limit microbial cell attachment, and reduce bacterial adhesion [24]. This effect arises from the amphiphilic nature of bacterial cell membranes and the presence of hydrophilic outer layers, such as lipopolysaccharides in Gram-negative bacteria, which diminish the ability of bacterial cells to interact with hydrophobic surfaces, thereby slowing down or even preventing bacterial colonization [25].

Moreover, copper (Cu) coatings are considered to be among the most promising materials for antimicrobial activity [26,27]. The development of the coating process involves terms such as deposition technique and structural design, with the aim of producing cost-efficient, functional products that are commercially available to all market sectors [28,29]. Moreover, the development of coatings and surfaces that can actively kill microbes is an important part of maintaining hygiene in hospital environments, and various coating methods have demonstrated the antimicrobial activity of Cu surfaces [30,31,32,33]. Graham et al. [33] obtained a durable, transparent, antibacterial Cu surface on glass, produced by thermal evaporation of ultra-thin metal films. The resulting Cu nanoparticle coating reduced *S. aureus* bacteria by more than 99.9%, while maintaining 70–80% transparency to visible light, making the material suitable for touch screen applications. Another study [34] confirmed that changes in magnetron sputtering conditions play a key role in determining both the microstructure and optical properties of coatings with graded functionality. The produced TiCu(Ag) thin films exhibited properties that allow for very effective adhesion and proliferation of human MRC-5 fibroblast cells. In addition, all TiCu(Ag) alloy compositions tested exhibited strong antibacterial activity, leading to the inhibition of *Pseudomonas aeruginosa* and *S. aureus* growth. The deposition took place using mono-alloy targets of Ti, Cu, and Ag, each featuring a 3″ diameter and 99.99% purity [34]. Qin et al. [15] produced Ti–Cu coatings using a combination of HPPMS and DC, followed by vacuum temperature treatment at 300, 400, and 500 °C. Ti, Cu, and CuTi_3_ were mainly formed in the coatings before annealing, while Ti_3_O, Cu_2_O, and CuTi_3_ were the main annealing products. Thin-film metallic glasses (TFMGs) were deposited on P-type (100) Si and AISI 304 stainless steel substrates using High-Power Impulse Magnetron Sputtering (HiPIMS) technology. The Zr, Cu, and Ti targets (45.3 × 17 cm^2^) [14] were used in the production.

Although many studies reported results on coatings with high Cu and/or Ti content produced by magnetron sputtering, these coatings were generally deposited on small-area substrates under laboratory-scale conditions and designed for medical applications, such as implant-related uses [32,35,36,37]. The significant aspect of novelty in this research was the use of multi-alloy targets in an industrial magnetron sputtering line for the deposition of transparent coatings onto large-area glass panes (up to production line capabilities, i.e., 2.5 m long and 1.20 m wide). Moreover, based on literature sources, a large number of multi-composite targets with various metal compositions and percentages were strategically combined, produced, and tested for the first time. This work presents the relationships between microstructure, optical transparency (arising from the thickness of the coatings), durability (in terms of chemical and abrasion resistance), wettability, and antibacterial performance under industrially relevant conditions.

Many studies have evaluated coatings with high Cu and Ti content using contact times ranging from 2 to 24 h and incubation at 37 °C. These studies have typically employed either colony-forming units (CFU) enumeration or biofilm/adhesion assays against *E. coli*, *S. aureus*, and related bacterial strains. These experimental conditions are generally considered to be representative of the physiological environment relevant to surface applications [35,38,39]. Greenhouse crops such as tomatoes and leafy vegetables generally prefer moderate temperatures, with optimal growth occurring below 30 °C. Tomatoes exhibit reduced fruit set when daily mean temperatures exceed 30 °C, while leafy vegetables such as lettuce achieve maximum yields under slightly cooler conditions of around 22–30 °C with appropriate humidity control [40,41,42]. This study evaluated the antibacterial properties of coatings under conditions representative of a greenhouse environment by conducting experiments at moderate temperatures of around 28 °C. This is an original approach, as most experiments nowadays are conducted at temperatures characteristic of the human body (approximately 37 °C) [16,32,43,44,45].

To the best of our knowledge, this is the first time that different Cu- and Ti-based coatings have been obtained from multi-alloy targets on an industrial scale. A deposition process under oxygen, with or without argon, has been directly performed on a glass surface of at least 0.25 m^2^, using different targets, e.g., Cu90 Zn10, Cu90 Sn10, and Cu80 Ti20. The antibacterial activity of these coatings has not yet been studied under conditions representative of a greenhouse environment, with experiments being conducted at moderate temperatures of around 28 °C.

The goal of the research was to develop efficient technology for producing glass surfaces with bactericidal coatings on an industrial scale using multi-alloy targets. To select those to be used in further greenhouse experiments, we have tested different technical parameters for coating production and various coating contents, as well as their antibacterial activity at moderate temperatures.

## 2. Materials and Methods

### 2.1. Preparation of Glass Coatings

**Figure 1:**
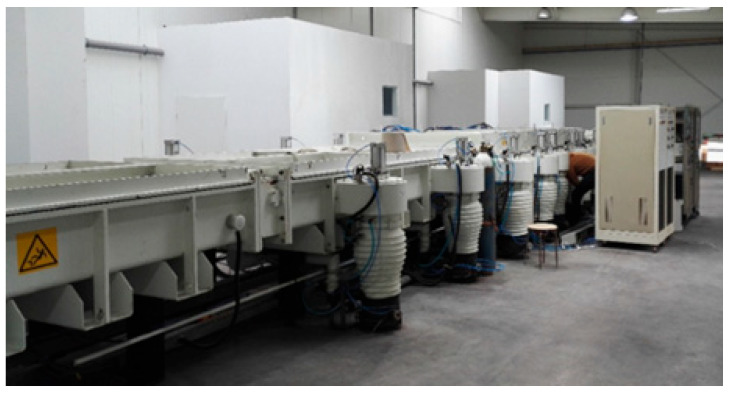
An industrial horizontal magnetron sputtering line by D.A.Glass Sp. z o.o.

A batch of samples with magnetron sputtered coatings was prepared on a diffused glass (a chemically etched glass with an effective light-spreading property). An industrial magnetron sputtering line (Figure 1) (TS-3000CJLW (V11.05), a horizontal-type product line produced by China Guangdong PVD Metallizer Co., Zhaoqing, China, in 2011) was used for this purpose. The main advantage of this line compared with laboratory coaters is the possibility to perform a process on a glass surface up to 2.5 m long and 1.20 m wide.

The following targets were used to prepare the coatings—series no. II–XV (Table 1). The process was carried out under oxygen or oxygen/argon conditions with a DC switching Dora Power Supply (30 kW, 3-modular, produced by Dora Power System in Wilczyce (Poland)). The roller speed and transfer amount were adjusted to receive glass direct transmittance not lower than 85% (D5) and 88% (D8) (Table 1).

The optical properties of the samples were measured using a D.A.Glass Sp. z o.o. spectrophotometer (Wageningen UR Greenhouse Horticulture, serial number 003/2009, Wageningen, The Netherlands) with an Ulbricht sphere (1 m diameter) in the range of 400–700 nm (photosynthetically active radiation; PAR), according to the norm NEN 2675:2018 Greenhouse glass—Determination of optical properties of greenhouse covering materials and screens [46], which is dedicated to greenhouse application. Both direct and hemispherical (measured at different angles of incident light) transmittance on the surface of 0.5 by 0.5 m samples were defined.

The samples were further examined using an FEI TITAN 80-300 scanning-transmission microscope (STEM) (FEI Company, Hillsboro, OR, USA) equipped with an energy-dispersive X-ray spectroscopy (EDS) detector for chemical composition microanalysis and an enhanced-brightness Field Emission Gun emitter. A Quanta 3D 200i microscope (FEI company, Hillsboro, OR, USA) equipped with an ion gun for micromachining materials and a platinum source was used for sample preparation. The study of thin films on glass substrates, which do not conduct electrical charge, required a specialized methodology involving the preparation of samples using the focused ion beam method. For this purpose, a procedure was used to prepare the surface of the samples using current parameters to obtain samples (laths) with a thickness of <100 nm. Prior to the cutting process using a gallium ion beam, a thin layer of gold was sputtered onto the surface of the material to ensure the dissipation of electrical charge during micromachining. In addition, a conductive connection was made between the test surface and the Quanta 3D 200i microscope table using self-adhesive Cu tape prior to proceeding.

The characterization included measurements of the coating thickness and determination of their phase composition using High-Resolution Transmission Electron Microscopy (HRTEM) (FEI company, Hillsboro, OR, USA) and Fast Fourier Transform (FFT). For each sample, 10 measurements of the coating thickness have been performed.

### 2.2. Bacteria Cultures and Test for Biocidity

**Figure 2:**
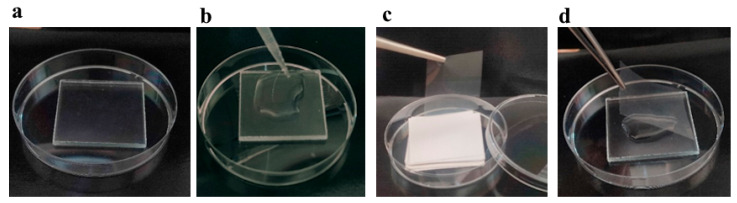
(**a**) Squares (50 mm × 50 mm) made of coated glass; (**b**) bacterial suspension on the surface of coated glass; (**c**) sterile polypropylene foils, separated by pieces of paper for autoclaving; (**d**) covering bacterial suspensions with foil to protect the suspension from evaporation.

The antibacterial activity of the coated glass was evaluated against microorganisms: *E. coli* (PCM 2560), *M. luteus* (PCM 525), *S. aureus* (PCM 2602) obtained from the Polish Collection of Microorganisms (PCM) and *P. syringae* pv. tomato (IOR2146) obtained from the Institute of Plant Protection—National Research Institute in Poland. All bacteria were maintained on tryptic soy agar (TSA) medium at 4 °C and deposited in the Department of Industrial and Environmental Microbiology, UMCS, in Lublin. Squares (50 mm × 50 mm) made of coated glass were sterilized with 70% ethanol and placed in a sterile Petri dish (Figure 2a). Bacterial suspensions (1 mL) with a density of 10^6^ mL^−1^ of cells, corresponding to the same number of CFU, were inoculated onto the surface of the coated glass (Figure 2b) and covered with sterile polypropylene foils (about 48 mm × 48 mm, 50 µm thick) (Figure 2c,d). The CFU of the inoculum of each bacterium was also verified on TSA medium. The experiment was conducted in a phytotron chamber (Biogenet^®^, Quito, Ecuador) under two lighting conditions: with light (L) (LED tube 1200 mm, 15.5 W, 865 T8, light, about 1000 lux, spectral characteristics: 400–750 nm) and without light—dark (D) at 28 °C for 24 h. After incubation, the foils from the top of the tested surfaces were gently removed and the bacterial suspensions collected from the coated glass were sown on TSA medium. To detect the bactericidal potential of the tested nanolayers, the CFU was compared to the number of colonies grown at the same time on the control (which is diffuse glass without any coating). The assays were carried out in triplicate.

### 2.3. Durability, Hydrophilicity and Anticorrosion Analysis

Based on the results obtained, the three most effective coatings were selected (metal % in the target): (II) Cu90 Sn10 (D5), (III) Cu90 Zn10 (D5) and (IX) Cu80 Ti20 (D5) for further tests. The abrasion resistance test (according to report 6688/AT/1/89) was performed using a Taber Surface Analyser (Model 5155) produced by Taber Industries, North Tonawanda, NY, USA, with the following parameters: abrasion cycles, 200; type of abrasive wheels, CS-10; rotation speed, 60; suction force, 70%; load, 2 × 2.45 N. Before starting, samples were conditioned for 48 h under the following conditions: air humidity, 70 ± 5%; temperature, 23 ± 2 °C. Prior to each cycle, wetting of the sample surface with demineralized water in the form of a “water mist” was used to mimic greenhouse conditions as effectively as possible. In the test, three selected coatings and the control were subjected to abrasion cycling according to the scheme (10/90/100), i.e., 10 abrasion cycles were carried out, followed by another 90 and another 100 cycles, making a total of 200 abrasion cycles (Appendix A).

The anti-corrosion test (according to report LL/261/2020) was performed in a brine aging chamber under exposition to neutral salt spray at 25 °C. According to ISO 9227 [47], the experimental conditions were a 36h exposure to a mist of NaCl (pH = 7) at a concentration of 50 g/L ± 5 g/L. The coated samples were removed from the chamber, left to dry, and then washed with running water, rinsed with distilled water, and dried with a stream of air. The samples were visually inspected for corrosion (red-orange points). For the same coatings, the degree of wettability was measured using the PG-X+ Pocket Goniometer (Gardco, Columbia, MD, USA) was defined. Each sample was measured 5 times with a droplet volume of 10 µL.

### 2.4. Statistical Analysis

All statistical analyses were performed using RStudio (2025.05.1+513). Bacterial counts were first tested for normality using the Shapiro–Wilk test. Since the assumptions of parametric tests were met, differences between materials were assessed using one-way ANOVA followed by Tukey’s HSD post hoc test (α = 0.05). Confidence intervals (95%) for group means and Tukey’s comparisons were extracted using the emmeans package. Compact letter diagrams (CLDs) were generated using the multcompLetters4() function and are shown above the bars on each graph. Principal component analysis (PCA) was performed to explore patterns and relationships among measured variables. PCA was computed on the standardized data (variables were centered and scaled to unit variance) using prcomp() in RStudio. The number of retained principal components was chosen based on the scree plot (percentage of explained variance) and interpretability. Variable contributions, loadings, and individual scores were visualized using biplots and contribution plots.

## 3. Results and Discussion

### 3.1. Manufacturing and Characterization of Nanolayer Coated Glass

Coated glass samples were successfully deposited on 0.5 m × 0.5 m glass pieces with an industrial magnetron sputtering line (Figure 1), according to the process parameters in Table 2, including amperage (A), roller speed (m·min^−1^), the reactive and/or inert gas used, and gas flow (cm^3^·min^−1^). It is noteworthy that, considering the further stages of the research, in which the construction of experimental greenhouses was planned, it was the first time that glass up to 2 m by 0.9 m was coated with the same industrial line. To obtain samples of coated glass, 14 targets with different chemical compositions were used, with a possible broad spectrum of antimicrobial activity.

The aim of the glass manufacturing process was to obtain two samples from each target: one of a direct transmittance not lower than 88% (D8), and a second with a value not lower than 85% (D5). The anticipated value of direct transmittance not lower than 88% ensures the lowest possible light loss of PAR, which is crucial for the proper growth of plants. According to numerous researchers, greenhouse covering materials should be as transparent as possible to solar radiation. An increase of approximately 1% in material transparency typically leads to a corresponding 1% increase in crop yield [48,49]. Our coatings (samples D8) exhibited a decrease of only ca. 2% in PAR compared to the control diffuse sample (without any coating). They were investigated further for eight weeks in experimental greenhouses, where tomatoes were successfully cultivated until harvest time (data not published). This fact clearly confirms that the optimal light conditions for cultivating tomatoes have been achieved. However, yields also depend on CO_2_ concentration and air temperature. It is worth mentioning that excessive radiation can also hinder photosynthesis, which is why shading is necessary in summer (to reduce plant overheating), and artificial lighting is used in winter, especially in northern regions, to increase radiation levels [49,50]. In our experiment, a lower value of 85% was chosen to achieve different coating thicknesses and potentially enhance the antibacterial potential. This assumption was fulfilled for each coating obtained from all investigated targets (II–XV). The results of direct and hemispherical transmittance were collected in Table 2. Such obtained samples were further characterized to define their chemical composition using STEM coupled with an EDS detector. The coating thickness and phase were determined using HRTEM.

**Figure 3:**
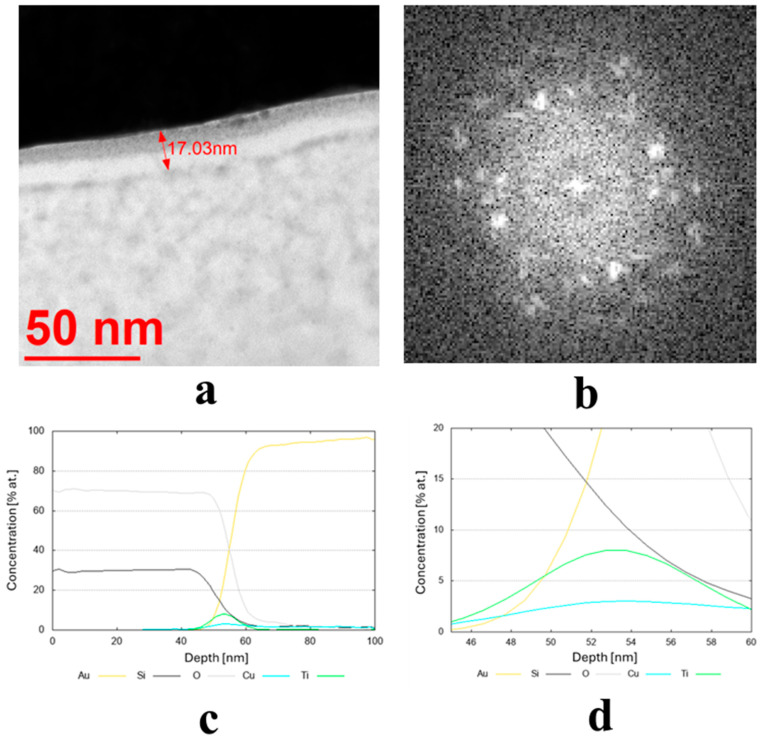
(**a**,**b**) Microstructure (TEM bright-field) image of the sample obtained from the target (IX) Cu80 Ti20 (D5) along with an FFT image showing reflexes from a crystalline structure and (**c**,**d**) chemical composition profiles (at. %) across the coating.

The results of the structure tests for selected samples are presented in Figure 3. Table 2 shows the average thickness measurement results, along with their standard deviation and phase composition for all samples. The average coating thickness ranged from 3.9 to 59.3 nm, as obtained for coatings made from Cu80 Ni10 Fe5 Mn5 and Cu70 Zn25 Ni5 targets, respectively. The most common coatings among the remaining samples had an average thickness ranging from 15 to 25 nm. They had a diverse crystallographic structure. Seven of them were found to have an amorphous structure, and seven a crystalline structure (Table 2).

**Figure 4:**
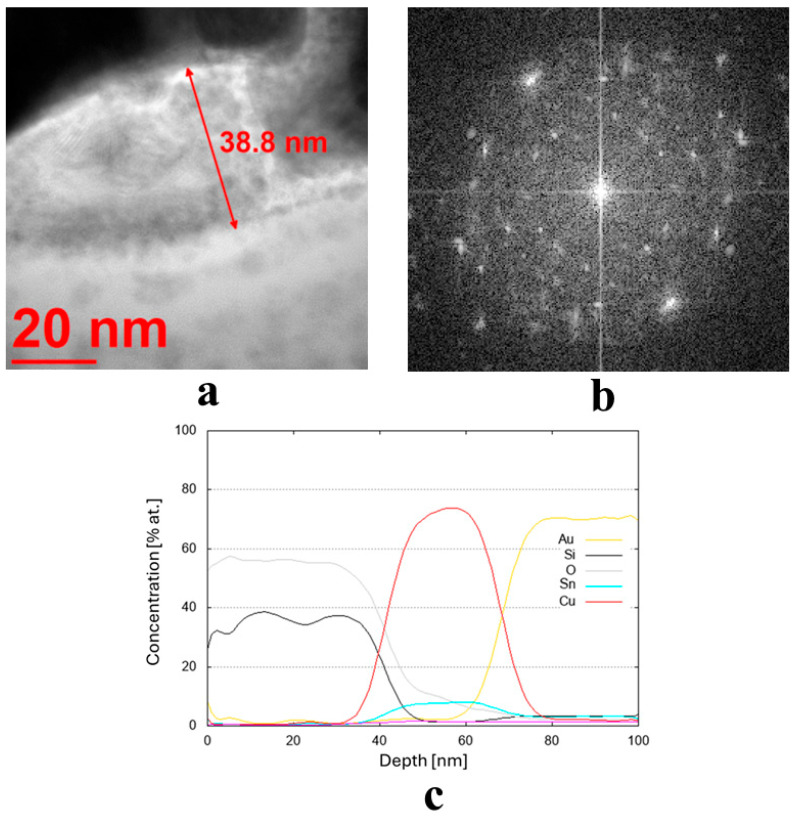
(**a**,**b**) Microstructure (TEM bright-field) image of the sample obtained from the target (II) Cu90 Sn10 (D8) along with an FFT image showing reflexes from a crystalline structure and (**c**) chemical composition profile (at. %) across the coating.

**Figure 5:**
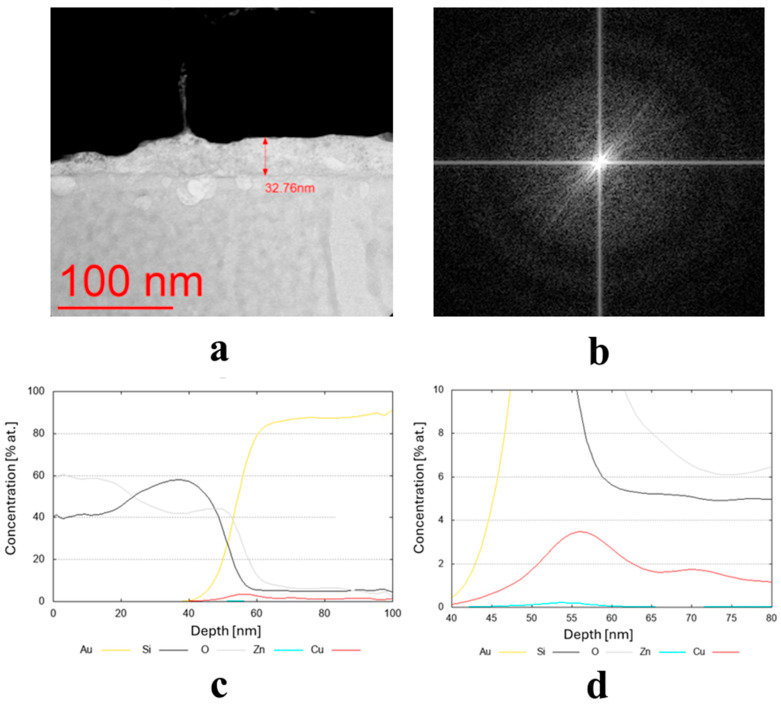
(**a**,**b**) Microstructure (TEM bright-field) image of the sample obtained from the target (III) Cu90 Zn10 (D8), along with a FFT image showing an amorphous (no crystalline reflexes) structure, and (**c**,**d**) chemical composition profiles (at. %) across the coating.

The transmission electron microscopy (TEM) bright-field images for the coatings obtained from the targets (IX) Cu80 Ti20 (D5), (II) Cu90 Sn10 (D8), and (III) Cu90 Zn10 (D8) are shown in Figure 3a, Figure 4a and Figure 5a, respectively. The sample sputtered from the target Cu80 Ti20 had the lowest thickness of around 17 nm, whereas the remaining coatings had thicknesses above 30 nm. The FFT images obtained from the HRTEM images confirm that the samples obtained from the targets (IX) Cu80 Ti20 and (II) Cu90 Sn10 were characterized by a crystalline structure, as indicated by the bright reflections in Figure 3b and Figure 4b. The amorphous characteristic of the sample produced from the target (III) Cu90 Zn10 coating is also evidenced in Figure 5b by a featureless FFT image. Amorphous coatings lack crystalline phases, resulting in a uniform, dense structure. Due to their ultra-smooth surface morphology without grain boundaries, they exhibit specific antibacterial properties that effectively inhibit bacterial adhesion. Furthermore, the addition of bactericidal ions (e.g., Cu^2+^, Cu^+^) further enhances this effect. In addition, amorphous coatings could provide long-lasting antibacterial functionality through the controlled release of metal ions [34].

Due to the very low thickness of the coatings, the exact measurement of their chemical composition was not possible. Although the studies were conducted using TEM, the lamella retains a measurable thickness (~100 nm) and volume, and therefore the contribution from the surrounding region remains significant. For this reason, line profiles were performed across the coatings’ thickness, indicating a semi-quantitative concentration of particular elements. These profiles are shown in Figure 3c,d, Figure 4c, and Figure 5c,d for the three discussed coatings and correspond well to the elements used to produce the cathodes. It is noteworthy that the signal from Au corresponds to the protective gold layer deposited on the coating before sample preparation. Signals from Si arise from silicium oxide (SiO_2_), which is present in the glass substrate.

The literature states that Cu can indeed act as a crucial antibacterial agent [27,51]. In fact, a single-phase film with a homogeneous surface distribution of Cu and/or Ag can be highly effective in protecting the surface against bacteria while maintaining high biocompatibility, thanks to the concurrent homogeneous distribution of TiO_2_ [34]. In another article, the cross-sectional TEM micrographs and corresponding EDS provided evidence that Ti is a major chemical element on the surface and interfaces of the silicon substrate. The Ti–Cu coatings were annealed at 500 °C. The bulk of the coatings contained Cu. The coatings were deposited on silicon wafers. No microbiological investigations were conducted with these materials [15]. Tang et al. [14] revealed an amorphous coating on the multilayer due to the high sputtering efficiency of Cu. The multilayer growth mechanism was dominated by Cu ions. Bacterial adhesion was decreased by the smooth surface and high hydrophobicity. The high-Cu-content samples were lethal to the bacteria, exhibiting a 97.6% reduction of *E. coli,* far exceeding that of SUS304 stainless steel. The tested two-layer coatings obtained by Markowska-Szczupak et al. [52] were characterized by various combinations of Cu, Au, and Ag layers. The authors found that antimicrobial activity is equal to or greater than that of single-layer coatings. On the other hand, the highest antibacterial effect (reduction of log CFU/mL) was observed for the Au/Cu and Ag/Cu coatings, in which the top layer was formed by Cu. This property was checked against *E. coli* and *Staphylococcus epidermidis.* The conclusions described above are congruent with our results, which indicate Cu species as the main antibacterial agent. It is important to remember that a combination of different mono-alloys was used to obtain different chemical compositions of the final coatings. In this study, various Cu-based coatings obtained from multi-alloy targets (for the first time on an industrial scale), such as Cu90 Zn10, Cu90 Sn10, and Cu80 Ti20, were applied directly on the glass surface to test their bactericidal effect. The use of multi-foot targets allows the sputtering process to be carried out in a single step, saving energy costs and increasing process efficiency. The functionality of the coatings prepared in this way was tested in both in vitro experiments (presented in Section 2.2 of this manuscript) and semi- and large-scale experiments (the results of these experiments will be presented in a separate article).

### 3.2. Antibacterial Effect of Coated Glass

**Figure 6:**
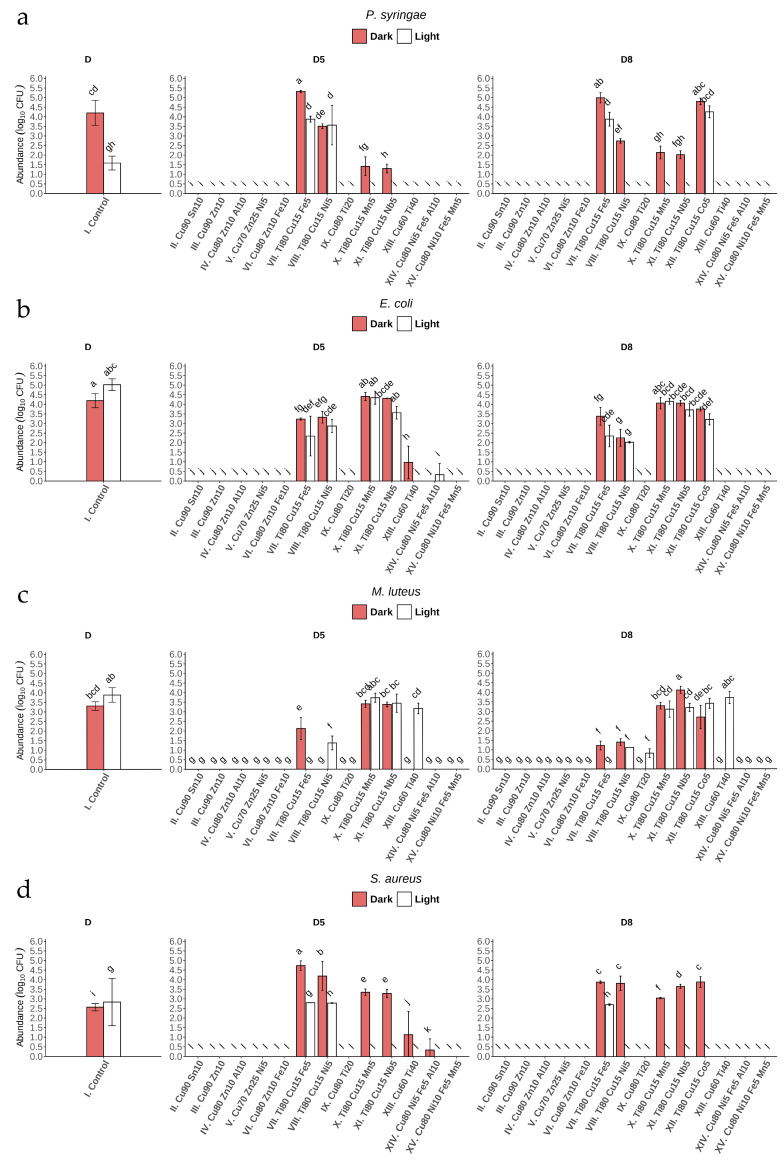
Number of bacteria (Log_10_ CFU mL^−1^) after 24 h of incubation on coated glass (I–XV) in light and dark conditions for: (**a**) *P. syringae*, (**b**) *E. coli*, (**c**) *M. luteus*, and (**d**) *S. aureus.1* Bars represent mean ± SD (n = 3). Letters denote statistically homogeneous groups based on Tukey’s HSD post hoc test (*p* < 0.05). One-way ANOVA: F(57,116) = 149.7, *p* < 2 × 10^−16^.

After 24 h of incubation of bacterial suspensions on the surface of glass coatings No. II, III, IV, V, VI, and XV (under light and dark conditions), total growth inhibition was observed (Figure 6a–d) (Appendix B). It is worth noting that these coatings have the highest Cu content compared to the others, and the antibacterial effect was observed for these coatings regardless of the value of direct transmittance: 88% (D8) and 85% (D5). Incubation of the bacterial suspensions on glass coatings No. XIII and XIV also completely inhibited the growth of most of the bacterial strains studied or resulted in a statistically significant decrease in growth compared to control, with the exception of the abundance of *M. luteus* after incubation on XIII (D5, D8) under light conditions (Figure 6c) (Appendix B).

Copper is a well-known antimicrobial metal ion, renowned for its ability to combat microbes through several potent mechanisms. In the environment, Cu can generate reactive oxygen species (ROS), such as hydrogen peroxide and hydroxyl radicals, through redox reactions. These ROS have oxidizing potential, which can disrupt microbial cell membranes, damage proteins and DNA, and interfere with critical cellular functions [31,51]. In view of the results obtained, it is necessary to verify the mechanism of metal ion release in our further research. Such analyses are particularly important in the case of coatings with a high Cu content, which exhibit clear antimicrobial activity. Similarly to Cu, zinc oxide (ZnO) also generates ROS in the environment, leading to oxidative stress and damage to bacterial cells [53]. Zinc ions can penetrate cells, disrupt membrane integrity, and interfere with cellular processes [54]. In search of an effective method of developing long-lasting coatings against bacterial cells using Zn and Sn oxides, the bactericidal activity of coatings containing 4 and 6 wt% of these oxides was evaluated using a quantitative method with an exposure time of 24 h [55]. It was found that the number of colony-forming units (CFU) decreased by 80–90% compared to the applied inoculum (1·10^7^ cells/mL^−1^) after this time, which is comparable to the antibacterial activity under conditions of severe bacterial contamination. The combined action of two or more coating components (e.g., Zn and/or Sn) can make Cu highly effective against a wide range of bacteria, viruses, and fungi [31,56]. Moreover, Cu has been found to interfere with biofilm formation, which plays a crucial role in bacterial resistance. Additionally, Gomes et al. [57], in in vitro experiments, showed that Cu ions disrupt the extracellular matrix of bacterial single- and dual-species biofilms, leading to high reductions of bacterial abundance (about 4 log CFU per cm^2^).

The microstructure of multicomponent coatings is a key factor influencing the activity of metal ion release. Amorphous coatings exhibit a smoother surface and greater structural stability, which reduces corrosion rates and limits ion release compared to crystalline coatings [58]. It is worth noting that in our studies, most coatings with high Cu content and strong antibacterial properties possess an amorphous structure (Table 2). For series II–VI, IX, and XIII–XV sputtered under oxygen (without argon), the metal oxides are expected to be major chemical species of the coatings; however, this must be confirmed in-depth with X-ray diffraction (XRD) analysis. This microstructure enables slower and more controlled release of particles from the surface while simultaneously inhibiting bacterial cell adhesion due to the smooth surface morphology. In contrast, in crystalline coatings, elements with experimentally confirmed biocidal activity, such as Cu or Ag, exhibit higher mobility, which affects both the mechanical integrity of the coating and leads to less controlled ion release [59,60]. It is noteworthy that in this study, almost all coatings (except XI) with high Ti content (Ti80) are crystalline (Table 2). Consequently, the release of particles from their surface may be less controlled, resulting in strong antibacterial activity; however, this structure is more favorable for biofilm formation.

In our investigations, exposure to glass coated under artificial visible light conditions (400–750 nm) with Nos. X, XI, and XII also had a strong effect on *P. syringae* and *S. aureus,* almost always causing total growth inhibition (Figure 6a,d). This bactericidal effect can be attributed not only to the effect of Cu, but in particular to the high Ti content of the three types of glass coating mentioned above. Importantly, this effect was not observed for the other two strains (Figure 6b,c). The Ti–Cu thin films obtained by Mahmoudi-Qashqay et al. [16] with various elemental percentages—Ti81–Cu19, Ti49–Cu51, Ti29–Cu71, and Ti14–Cu86—caused significant inhibition of bacterial growth (up to 99.9% CFU of bacteria mL^−1^) after 2 h of contact. Additionally, field emission scanning electron microscopy (FESEM) images confirmed the destruction of the cell membrane of both bacterial strains (*E. coli* and *S. aureus*) after only 30 min of exposure to Ti–Cu films. The findings of our study suggest that light plays an important role in the bactericidal effect of glass coatings containing Ti, which, in the form of titanium dioxide, might act as an efficient photocatalyst [61,62,63]. Further investigations are needed to determine which titania species (anatase and/or rutile, amorphous titania, and/or Cu-doped titania) should be examined. Due to the fact that the light source in the phytotron chamber emits light in Vis range, UV absorption in the potentially formed crystalline TiO_2_ must be excluded.

**Figure 7:**
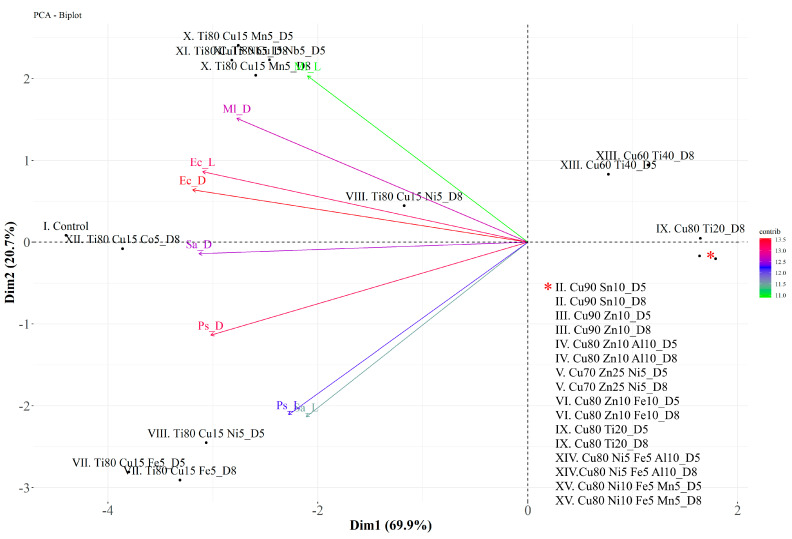
Biplot of the first (Dim 1) and second (Dim 2) principal components of the principal component analysis (PCA) of four bacterial strains (*P. syringae—*Ps, *E. coli—*Ec, *M. luteus—*Ml, and *S. aureus—*Sa) incubated on coated glass (II–XV) in light (L) and dark (D) conditions. * refers to the list of coatings assigned to two points in the bottom right quadrant of the chart.

In PCA, about 90.6% of the variation is explained by the first two eigenvalues together (Dim 1: 69.9% and Dim 2: 20.7%) (Figure 7). The variables EcD, EcL, and PsD contribute most to dimensions 1 and 2. The positively correlated variables are Ec with Ml and Ps with Sa. These results are presented in Figure 7. Similar individuals are grouped together in the same quadrant on the plot, e.g., II–VI (both D5 and D8), IX (except D8), and XIV–XV. Taking into account the clearly demonstrated antibacterial activity of coatings with the highest Cu content against all the microorganisms tested, as well as the possible additional mechanism of antimicrobial action of glass coatings containing Ti, the three coating glasses produced from the targets—(II) Cu90 Sn10, (III) Cu90 Zn10, and (IX) Cu80 Ti20—were selected for further research as the most efficient antibacterial agents.

### 3.3. Evaluation of Durability, Hydrophilicity, and Anticorrosion Properties

It is of high importance to validate the lifetime of the applied coatings, especially since they will be applied in further research in experimental greenhouses. Therefore, some durability tests were performed (Table 3).

After 10 abrasion cycles, no changes were observed on the surfaces of the analyzed coated glass samples (Nos. I: control, II, III, and IX). After another 90 cycles, abrasion marks were observed on the reference samples. After another 100 cycles, some abrasion marks were observed on the surfaces of the coated glass samples (Appendix A). The most visible effects were observed on the control samples, with a decrease for each of the coated glasses (Nos. II, III, and IX). The least visible traces were observed on samples marked I (obtained from Cu90 Sn10 target). Moreover, the traces observed on samples III (Cu90 Zn10 target) and IX (Cu80 Ti20 target) were significantly smaller compared to the control sample (I) and were therefore negligible (Table 3). The presented results prove that the selected coated glasses are more resistant to abrasion (by the above method) than the control glass without coating. In other words, they protect the glass from the mechanical factor of abrasion. This means that the selected coatings may be applied in further research in an experimental greenhouse.

The results of an anti-corrosion test according to ISO 9227 (with neutral sodium chloride spray) indicated minimal traces of corrosion (in the form of red-orange dots) (Table 3) (Appendix C). Magnetron-sputtered CuO coatings, especially when combined with other oxides or engineered surface features, offer robust corrosion resistance and mechanical durability. These properties make them suitable for applications requiring long-term protection against both mechanical impact and corrosive environments [64]. In a study on a potential dual-layer implant coating, with ZnO as the bottom layer and CuO as the top layer, developed by controlled magnetron sputtering, Zhang et al. [65] showed improved surface morphology and roughness, resulting in high corrosion resistance (corrosion potential up to −0.261 V), good mechanical durability, and enhanced protective efficiency. Similarly, a study on the production of an antibacterial, superhydrophilic TiO_2_–CuO_y_ nanocoating on a Ti sheet by reactive magnetron sputtering revealed that the surface of the TiO_2_–Cu_2_O coating showed very good corrosion resistance and mechanical durability [66]. Our results testify to the very good resistance of the selected coated glass as well as the of the control glass. A very good resistance to abrasion and glass corrosion is offered by our coatings, probably due to the protective properties of Ti, Cu, Zn, and Sn oxides.

The reference glass, i.e., diffusion glass without any coating (No. I), showed a wetting angle of 34° (Table 3). Three selected coated glasses produced from targets—No. III Cu90 Zn10, No. IX (Cu80 Ti20), and No. II Cu90 Sn10—led to an increase in the wetting angle to 73, 85, and 89°. Mahmoudi-Qashqay et al. [16] presented antibacterial binary Ti–Cu thin films with amorphous structure, obtained by co-current sputtering (with different percentages of elements). Moreover, similar to our study, the wetting angle was about 80° for the higher percentage of Cu in the thin films, indicating a tendency toward hydrophobicity. Hydrophobicity is commonly assessed by the water contact angle (WCA). Surfaces with WCA < 90° are classified as non-hydrophobic; however, those with WCA around 80–90° (such as those obtained in our study) exhibit slight hydrophobicity [67,68]. Hydrophobic coatings effectively repel water, thereby reducing bacterial adhesion. This property also decreases the adhesion of dust and other particulate contaminants, enabling their easy removal by water droplets or by an air stream [24,25]. A hydrophobic coating that reduces maintenance requirements in greenhouse environments makes this glass an attractive solution for creating greenhouses that do not require frequent maintenance. However, in humid environments such as greenhouses, hydrophobic surfaces can promote the formation of stable microdroplets. These droplets, especially when they form around clusters of bacteria, create local microenvironments with higher water potential, which significantly increases bacterial survival compared to drier conditions. As the hydrophobicity of the surface increases, the droplets take on a more compact, spherical shape, which leads to a reduction in the contact area (base) and an increase in height for the same volume, and consequently also to a greater tendency for them to detach and move with the air flow. This allows contaminants (including microbiological ones) to be transported to other surfaces in the greenhouse [69,70]. Taking this into account, the glass coatings exhibiting slight hydrophobicity, such as those obtained in our study, may be more advantageous for greenhouse applications.

## 4. Conclusions

In this study, the sputtering process was performed for the first time using multi-alloy targets to produce 14 glass coatings. They were composed of two to four components and incorporated varying concentrations of Cu, Sn, Zn, Al, Ni, Fe, Ti, Mn, Nb, or Co. These coatings were applied on glass surfaces equal to or larger than 0.25 m^2^, representing a novel achievement in this field. The coatings obtained from targets No. II Cu90 Sn10, No. III Cu90 Zn10, and No. IX Cu80 Ti20 (D5 and/or D8) were chosen for further research due to their promising antibacterial effect. Finally, the durability and corrosion resistance of the obtained coatings were confirmed.

As a result of the research conducted, an efficient technology employing multi-alloy targets has been developed, which appears suitable for industrial-scale implementation. A key factor in such a probable application is the production of the coatings as external glass nanolayers (directed towards the interior of the greenhouse), and the verification and characterization of their bactericidal activity to support subsequent experiments in greenhouses. Therefore, in-depth research into the mechanism of metal ion release will be required in the near future.

## Figures and Tables

**Table 1:** Sputtering conditions applied during coating deposition on large-area (0.5 m × 0.5 m) glass samples.

	Serie No.	Me% (In the Target)	Amperage [A]	Roller Speed [m·min^−1^]	Oxygen [cm^3^·min^−1^]	Argon [cm^3^·min^−1^]	Transfer Amount	Tdir ^(a)^ [%]	Them ^(b)^ [%]
D0	I	Control—diffuse glass without any coating						91.4	81.3
D5 ^(e)^	II	Cu90 Sn10	13 × 3	8	2 × 450 = 900	-	1	86.2	74.9
D8 ^(f)^			12 × 3	10	2 × 450 = 900	-	1	88.9	77.7
D5	III	Cu90 Zn10	13 × 3	11	2 × 450 = 900	-	1	84.7	76.5
D8			7 × 3	10	2 × 450 = 900	-	1	88.0	79.7
D5	IV	Cu80 Zn10 Al10	13 × 3	1.6	2 × 450 = 900	-	1	85.2	73.8
D8			13 × 3	8	2 × 450 = 900	-	1	88.1	76.6
D5	V	Cu70 Zn25 Ni5	10 × 3 ^(c)^	4	2 × 450 = 900 ^(d)^	-	1	85.0	73.2
D8			10 × 3	5.6	2 × 450 = 900	-	1	88.3	76.8
D5	VI	Cu80 Zn10 Fe10	10 × 3	4.4	2 × 450 = 900	-	1	84.6	73.8
D8			10 × 3	7	2 × 450 = 900	-	1	88.3	77.7
D5	VII	Ti80 Cu15 Fe5	10 × 3	0.8	450	450	3	85.8	73.6
D8			10 × 3	1	450	450	3	88.1	76.5
D5	VIII	Ti80 Cu15 Ni5	10 × 3	2	450	450	3	85.0	73.5
D8			10 × 3	4	450	450	3	88.8	77.8
D5	IX	Cu80 Ti20	10 × 3	1.2	2 × 350 = 700	-	1	85.1	73.6
D8			10 × 3	2	2 × 350 = 700	-	1	87.7	76.8
D5	X	Ti80 Cu15 Mn5	10 × 3	0.8	450	450	3	86.8	75.1
D8			10 × 3	0.6	450	450	5	88.3	77.2
D5	XI	Ti80 Cu15 Nb5	10 × 3	0.8	450	450	3	86.3	74.8
D8			10 × 3	1	450	450	3	88.1	77.1
D8	XII	Ti80 Cu15 Co5	10 × 3	1	450	450	3	87.6	76.7
D5	XIII	Cu60 Ti40	10 × 3	1.1	2 × 350 = 700	-	3	85.1	73.5
D8			10 × 3	0.8	2 × 350 = 700	-	1	88.0	77.0
D5	XIV	Cu80 Ni5 Fe5 Al10	10 × 3	1.2	2 × 400 = 800	-	1	85.0	73.7
D8			10 × 3	2	2 × 400 = 800	-	1	88.4	77.2
D5	XV	Cu80 Ni10 Fe5 Mn5	10 × 3	6	2 × 400 = 800	-	1	85.0	73.8
D8			10 × 3	8	2 × 400 = 800	-	1	88.4	77.1

^(a)^ Tdir—direct transmittance measured in the Vis range (photosynthetically active radiation, PAR: 400–700 nm); ^(b)^ Them—hemispherical transmittance measured under different angles of incident light (between 0 and 90°); ^(c)^ 10 × 3—means 10 A multiplied by 3 (because of the fact that power supply is 3-modular), giving 30 A; ^(d)^ 2 × 450—means that 450 [cm^3^·min^−1^] gas was fed through two dispensing tubes simultaneously; ^(e)^ D5 direct transmittance not lower than 85%; ^(f)^ D8 direct transmittance not lower than 88%.

**Table 2:** Results of structure tests including measurements of coating thickness and crystal structure of coatings.

	Serie No.	Me% (In the Target)	Coating Thickness [nm]	Standard Deviation [nm]	Crystal Structure
D0	I	Control—diffuse glass without any coating	41.0	7.6	-
D5 ^(a)^	II	Cu90 Sn10	16.7	7.6	Crystalline
D8 *^,(b)^			27.5	13.7	Crystalline
D5	III	Cu90 Zn10	18.8	3.5	Amorphous
D8 *			22.4	5.0	Amorphous
D5	IV	Cu80 Zn10 Al10	43.8	3.3	Amorphous
D8			8.6	2.9	Amorphous
D5	V	Cu70 Zn25 Ni5	24.7	3.6	Amorphous
D8			59.3	5.9	Amorphous
D5	VI	Cu80 Zn10 Fe10	43.1	12.2	Amorphous
D8			16.7	3.6	Amorphous
D5	VII	Ti80 Cu15 Fe5	21.4	1.0	Crystalline
D8			22.7	2.2	Crystalline
D5	VIII	Ti80 Cu15 Ni5	13.8	1.1	Crystalline
D8			17.6	3.5	Crystalline
D5 *	IX	Cu80 Ti20	15.7	4.8	Crystalline
D8			12.5	2.3	Crystalline
D5	X	Ti80 Cu15 Mn5	26.5	1.2	Crystalline
D8			24.5	1.6	Crystalline
D5	XI	Ti80 Cu15 Nb5	24.5	2.2	Amorphous
D8			22.4	2.8	Amorphous
D5	XII	Ti80 Cu15 Co5	22.6	3.0	Crystalline
D8			23.6	0.8	Crystalline
D5	XIII	Cu60 Ti40	24.6	2.1	Crystalline
D8			20.1	3.7	Crystalline
D5	XIV	Cu80 Ni5 Fe5 Al10	8.5	1.8	Amorphous
D8			6.7	2.3	Amorphous
D5	XV	Cu80 Ni10 Fe5 Mn5	3.9	0.9	Amorphous
D8			6.7	4.6	Amorphous

^(a)^ D5 direct transmittance not lower than 85%; ^(b)^ D8 direct transmittance not lower than 88%; * coatings selected for detailed TEM characterization.

**Table 3:** Results of durability, hydrophobicity, and anticorrosion assessments for selected coatings.

Serie No.	Me% (In the Target)	Durability *	Wettability/ Contact Angles [°] ***	Anticorrosion **
I	Control—diffuse glass without any coating	low	34 ± 6	1 point
II	Cu90 Sn10	high	89 ± 7	2 points
III	Cu90 Zn10	medium	73 ± 4	3 points
IX	Cu80 Ti20	medium	85 ± 5	1 point

* Scale of durability: 4–5—very intense abrasion traces of abrasion (low); 3–4—medium intensity abrasion traces (medium) 1–2—almost invisible abrasion traces (high). ** Scale of anticorrosion resistance (Neutral Salt Spray according to norm ISO 9227): 0–5 corrosion points (in the form of red-orange dots)/very good anticorrosion resistance; 5–10 corrosion points/medium abrasion resistance; >10 corrosion points/low abrasion resistance. *** Contact angles with standard deviation values from five measurements.

**Figure A1:**
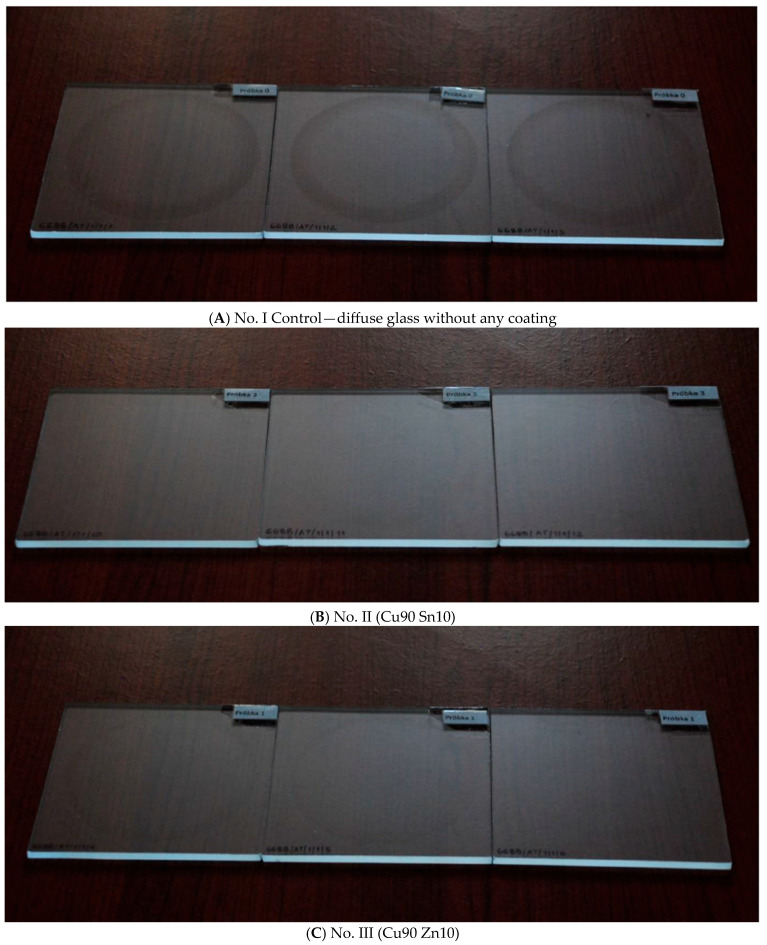

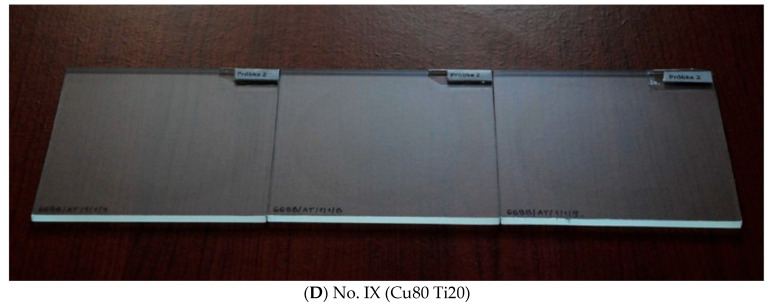
(**A**–**D**). Surface abrasion effects measured by the Taber Surface Analyzer (Model 5155) according to report 6688/AT/1/89 for three selected coatings.

**Figure A2:**
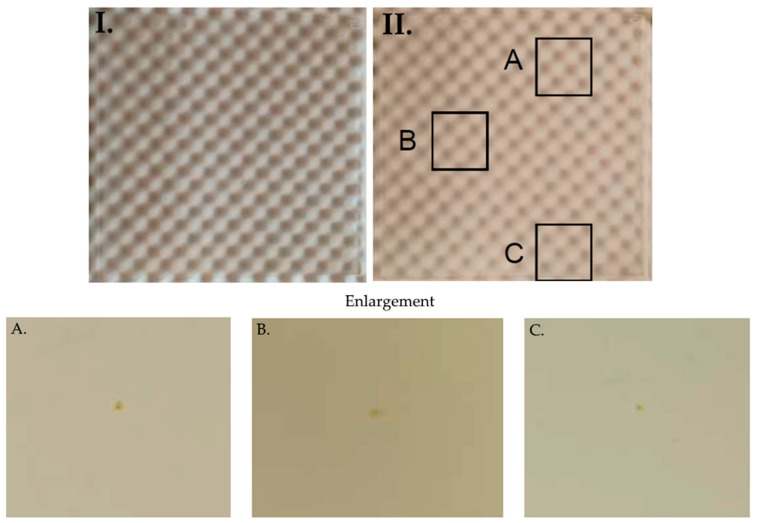
Coated glass sample No. III obtained from the target Cu90Zn10 before (**I**) and after corrosion tests (**II**), and enlargement of detail A, B, and C.

**Figure A3:**
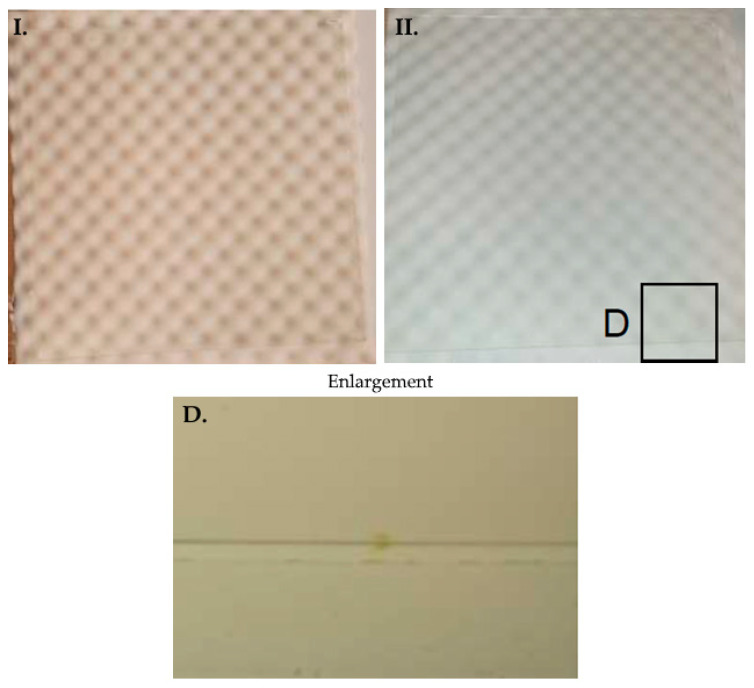
Coated glass sample No. IX obtained from the target Cu80Ti20 before (**I**) and after corrosion tests (**II**), and enlargement of detail D.

**Figure A4:**
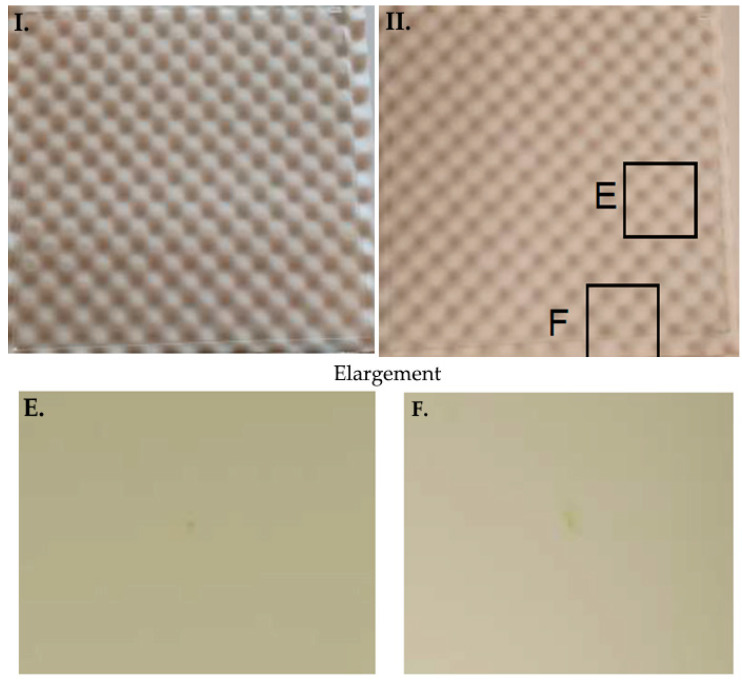
Coated glass sample No. II obtained from the target Cu90Sn10 before (**I**) and after corrosion tests (**II**), and enlargement of detail E and F.

**Figure A5:**
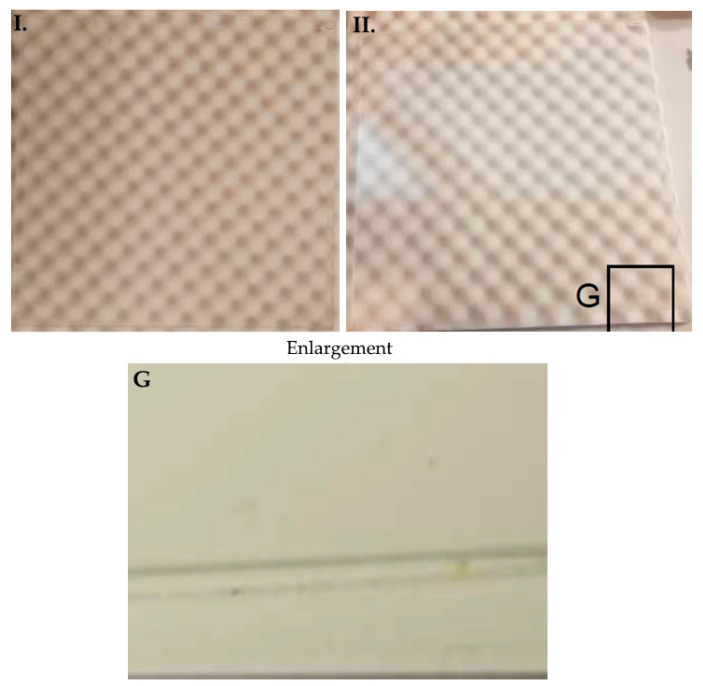
Control glass sample—diffuse glass without any coating before (**I**) and after corrosion tests (**II**), and enlargement of detail G.

## Data Availability

The original contributions presented in this study are included in the article. Further inquiries can be directed to the corresponding author.

## Appendix B

**Table A1:** Estimated marginal means (emmeans) of bacterial abundance (log_10_ CFU mL^−1^) for individual materials with 95% confidence intervals, obtained from one-way ANOVA followed by Tukey’s HSD post hoc test. Different letters indicate statistically significant differences between materials (*p* < 0.05).

Metal	Emmean	SE	df	Lower.CL	Upper.CL	Letters
Cu60Ti40D5_D *	1.99604 × 10^−15^	0.131275363	116	−0.260007398	0.260007398	i
Cu60Ti40D5_L **	−1.56417 × 10^−14^	0.131275363	116	−0.260007398	0.260007398	i
Cu60Ti40D8_D	−3.72185 × 10^−15^	0.131275363	116	−0.260007398	0.260007398	i
Cu60Ti40D8_L	2.48759 × 10^−14^	0.131275363	116	−0.260007398	0.260007398	i
Cu70Zn25Ni5D5_D	−2.6752 × 10^−15^	0.131275363	116	−0.260007398	0.260007398	i
Cu70Zn25Ni5D5_L	2.19439 × 10^−15^	0.131275363	116	−0.260007398	0.260007398	i
Cu70Zn25Ni5D8_D	−1.90051 × 10^−15^	0.131275363	116	−0.260007398	0.260007398	i
Cu70Zn25Ni5D8_L	3.05346 × 10^−16^	0.131275363	116	−0.260007398	0.260007398	i
Cu80Ni10Fe5Mn5D5_D	4.00571 × 10^−15^	0.131275363	116	−0.260007398	0.260007398	i
Cu80Ni10Fe5Mn5D5_L	−1.44256 × 10^−15^	0.131275363	116	−0.260007398	0.260007398	i
Cu80Ni10Fe5Mn5D8_D	4.86075 × 10^−15^	0.131275363	116	−0.260007398	0.260007398	i
Cu80Ni10Fe5Mn5D8_L	−2.4304 × 10^−15^	0.131275363	116	−0.260007398	0.260007398	i
Cu80Ni5Fe5Al10D5_D	−2.06523 × 10^−15^	0.131275363	116	−0.260007398	0.260007398	i
Cu80Ni5Fe5Al10D5_L	3.68052 × 10^−15^	0.131275363	116	−0.260007398	0.260007398	i
Cu80Ni5Fe5Al10D8_D	1.2153 × 10^−15^	0.131275363	116	−0.260007398	0.260007398	i
Cu80Ni5Fe5Al10D8_L	−5.07241 × 10^−15^	0.131275363	116	−0.260007398	0.260007398	i
Cu80Ti20D5_D	1.74345 × 10^−15^	0.131275363	116	−0.260007398	0.260007398	i
Cu80Ti20D5_L	−1.26054 × 10^−15^	0.131275363	116	−0.260007398	0.260007398	i
Cu80Ti20D8_D	1.22267 × 10^−15^	0.131275363	116	−0.260007398	0.260007398	i
Cu80Ti20D8_L	−3.43396 × 10^−16^	0.131275363	116	−0.260007398	0.260007398	i
Cu80Zn10Al10D5_D	1.40986 × 10^−17^	0.131275363	116	−0.260007398	0.260007398	i
Cu80Zn10Al10D5_L	3.31226 × 10^−16^	0.131275363	116	−0.260007398	0.260007398	i
Cu80Zn10Al10D8_D	−3.40328 × 10^−15^	0.131275363	116	−0.260007398	0.260007398	i
Cu80Zn10Al10D8_L	−2.36695 × 10^−15^	0.131275363	116	−0.260007398	0.260007398	i
Cu80Zn10Fe10D5_D	−2.11299 × 10^−15^	0.131275363	116	−0.260007398	0.260007398	i
Cu80Zn10Fe10D5_L	−2.85476 × 10^−15^	0.131275363	116	−0.260007398	0.260007398	i
Cu80Zn10Fe10D8_D	−2.33606 × 10^−15^	0.131275363	116	−0.260007398	0.260007398	i
Cu80Zn10Fe10D8_L	−3.0557 × 10^−15^	0.131275363	116	−0.260007398	0.260007398	i
Cu90Sn10D5_D	−3.74158 × 10^−15^	0.131275363	116	−0.260007398	0.260007398	i
Cu90Sn10D5_L	5.97023 × 10^−16^	0.131275363	116	−0.260007398	0.260007398	i
Cu90Sn10D8_D	−2.57648 × 10^−15^	0.131275363	116	−0.260007398	0.260007398	i
Cu90Sn10D8_L	−2.85494 × 10^−15^	0.131275363	116	−0.260007398	0.260007398	i
Cu90Zn10D5_D	6.40611 × 10^−15^	0.131275363	116	−0.260007398	0.260007398	i
Cu90Zn10D5_L	−3.74885 × 10^−16^	0.131275363	116	−0.260007398	0.260007398	i
Cu90Zn10D8_D	−5.96345 × 10^−16^	0.131275363	116	−0.260007398	0.260007398	i
Cu90Zn10D8_L	−1.76941 × 10^−15^	0.131275363	116	−0.260007398	0.260007398	i
Control ***_D	4.201697021	0.131275363	116	3.941689623	4.461704419	cd
Control_L	1.586697458	0.131275363	116	1.32669006	1.846704855	gh
Ti80Cu15Fe5D5_D	5.324921053	0.131275363	116	5.064913655	5.584928451	a
Ti80Cu15Fe5D5_L	3.876504906	0.131275363	116	3.616497509	4.136512304	d
Ti80Cu15Fe5D8_D	4.988829511	0.131275363	116	4.728822113	5.248836909	ab
Ti80Cu15Fe5D8_L	3.877865742	0.131275363	116	3.617858344	4.13787314	d
Ti80Cu15Mn5D5_D	1.418424168	0.131275363	116	1.158416771	1.678431566	gh
Ti80Cu15Mn5D5_L	−2.32161 × 10^−15^	0.131275363	116	−0.260007398	0.260007398	i
Ti80Cu15Mn5D8_D	2.137078925	0.131275363	116	1.877071527	2.397086322	fg
Ti80Cu15Mn5D8_L	−1.26211 × 10^−15^	0.131275363	116	−0.260007398	0.260007398	i
Ti80Cu15Nb5D5_D	1.301029996	0.131275363	116	1.041022598	1.561037394	h
Ti80Cu15Nb5D5_L	2.60913 × 10^−15^	0.131275363	116	−0.260007398	0.260007398	i
Ti80Cu15Nb5D8_D	2.02048416	0.131275363	116	1.760476762	2.280491558	fgh
Ti80Cu15Nb5D8_L	−4.66799 × 10^−16^	0.131275363	116	−0.260007398	0.260007398	i
Ti80Cu15Ni5D5_D	3.506810026	0.131275363	116	3.246802628	3.766817424	de
Ti80Cu15Ni5D5_L	3.562757474	0.131275363	116	3.302750076	3.822764872	d
Ti80Cu15Ni5D8_D	2.736396502	0.131275363	116	2.476389104	2.9964039	ef
Ti80Cu15Ni5D8_L	1.47649 × 10^−15^	0.131275363	116	−0.260007398	0.260007398	i
Ti8Cu15Co5D8_D	4.801949101	0.131275363	116	4.541941703	5.061956499	abc
Ti8Cu15Co5D8_	4.25542933	0.131275363	116	3.995421932	4.515436728	bcd

* D— in dark conditions; ** L— in light conditions; *** Control—diffuse glass without any coating.
